# hiPSC-Derived Cells as Models for Drug Discovery

**DOI:** 10.3390/ijms22168626

**Published:** 2021-08-11

**Authors:** Rivka Ofir

**Affiliations:** BGU-iPSC Core Facility, The Regenerative Medicine & Stem Cell (RMSC) Research Center, Ben Gurion University of the Negev, Be’er Sheva 84105, Israel; rivir@bgu.ac.il

More than 85% of pre-clinically tested drugs fail during clinical trials, which results in a long, inefficient and costly process, suggesting that animal models are often poor predictors of human biology [1]. The ability to perform research on humans is limited by the lack of physiologically relevant cells (especially the development and assessment of human brain cells and human heart cells). Currently, there are technologies to reprogram adult somatic cells (e.g., skin biopsy, blood cells, etc.) back into a pluripotent stage, termed induced pluripotent stem cells (iPSCs), and to differentiate pluripotent cells in vitro into many cell types of the body such as heart, muscle, brain cells, etc. [2]. These capabilities open a new era in human disease modeling [3].

The research topic of this issue is aimed at providing further context to the use of iPSC-derived cells (cardiomyocytes, fibroblasts, glial cells, neurons, astrocytes, brain microvascular endothelial cells and more) as disease models (“disease in a dish” models) for screening leads for drugs.

In this context, Trudler et al. [4], Lu Qian et al. [5], Rosner at al. [6], Zahumenska et al. [7] and Li et al. [8] reviewed recent models that further illuminate the potential of using iPSC-based platforms for drug discovery.

Wang et al. [9] describe strategies for assessing iPSC-derived cells’ therapeutic effects via transdifferentiation ability and exosomes through a paracrine mechanism. The review summarized the therapeutic effects of iPSC-derived exosomes on various disease models such as angiogenesis, cell proliferation and anti-apoptosis, with the hopes of improving their potential roles in clinical applications and functional restoration [9]. According to Tamo et al. [10], Induced pluripotent stem cell secretome (iPSC-CM) helps macrophages in tissue repair and regeneration. They identified Amyloid precursor protein (APP) and ELAV-like protein 1 (ELAVL-1), both present in the iPSC-CM, as the main players in regulating the function of macrophages in tissue repair. 

According to Vokner et al.’s [11] review, studies of Niemann–Pick disease Type C1 (NPC1) iPSCs-based models in comparison to to commonly used NPC1 models identify impaired autophagy as a central element in the pathogenesis of NPC1. 

Interestingly, iPSC-based models can also serve for screening potential drugs against complex diseases such as Parkinson’s disease [12], Alzheimer’s disease [13,14], Amyotrophic lateral sclerosis (ALS [15,16]) and for screening drug toxicity in iPSC 2D and 3D platforms [17,18]. 

The model of iPSC-derived cardiomyocytes with very long-chain acyl-CoA dehydrogenase deficiency (VLCADD), studied by Knottnerus et al. [19], implies that accumulation of fatty acid oxidation intermediates leads to cardiac arrhythmias. This study suggests that agents that will enhance fatty acid oxidation flux through increased mitochondria biogenesis or by inhibition of fatty acid transport into the mitochondria are potential drugs for VLCADD-CMs. 

iPSC-derived cardiomyocytes also serve as attractive models for dilated cardiomyopathy, such as propionic acidemia (PA), caused by mutations in either the PCCA or PCCB genes encoding both subunits of the mitochondrial propionyl-CoA carboxylase (PCC) enzyme [20] and in Coxsackievirus B3 (CVB3) infection [21]. 

Urea cycle disorders are enzymopathies resulting from inherited deficiencies in any genes of the cycle. Zabulica et al. [22] demonstrated the use of iPSCs of patients with a urea cycle defect for correcting their genetic mutation. In the edited cells, the defect was corrected, suggesting that this approach can serve as an in vitro model to advance the corrective cell-based therapy. 

To study the rare disease riboflavin transporter deficiency (RTD), Marioli et al. [23] used iPSC-derived neurons. This model can also shed light on the pathogenesis of neurodegenerative disorders. In these pathologies, the mitochondria do not function well. Among the tested antioxidants, EPI-743 restored the redox status, improved neurite length and ameliorated intracellular calcium influx into RTD motoneurons, suggesting that antioxidant supplementation may have a role in RTD treatment.

Among the applicative future goals in studying iPSCs is the potential to generate patient specific organs such as liver, hearth patch, etc. Olgasi et al. [24] describe the importance and potential of generating liver organs based on knowledge from iPSC tissue culture and emphasis its important implications for organ transplantation. Van Duinen et al. [25] established iPSC-based endothelial microvessels that closely mimic the process of angiogenesis in vivo and they develop a perfused 3D robust and scalable angiogenesis assay that is amenable for screening of anti-angiogenic compounds.

Pregnancy miscarriages have many unknown causes and are complex processes that require solution. Bohnke et al. [26] were able to mimic pregnancy complications associated with the enterovirus family that lead to miscarriages by infecting iPSC-derived primary germ-layer cells with coxsackievirus B3 (CVB3). Among iPSC-derived germ-layer cells, mesodermal cells were especially vulnerable to CVB3 infection. These cells can be considered as an in vitro platform for further consideration of members of the enterovirus family in the screening program of human pregnancies.

## Data Availability

Not applicable.
